# Elucidating causal relationships of diet-derived circulating antioxidants and the risk of osteoporosis: A Mendelian randomization study

**DOI:** 10.3389/fgene.2024.1346367

**Published:** 2024-06-07

**Authors:** Kexin Yuan, Xingwen Xie, Weiwei Huang, Dingpeng Li, Yongli Zhao, Haodong Yang, Xuetao Wang

**Affiliations:** ^1^ Gansu University of Chinese Medicine, Lanzhou, China; ^2^ Affiliated Hospital of Gansu University of Chinese Medicine, Lanzhou, China; ^3^ The Second People’s Hospital of Gansu Province, Lanzhou, China

**Keywords:** diet-derived antioxidants, oxidative stress, osteoporosis, bone mineral density, fractures, Mendelian randomization

## Abstract

**Background:**

Osteoporosis (OP) is typically diagnosed by evaluating bone mineral density (BMD), and it frequently results in fractures. Here, we investigated the causal relationships between diet-derived circulating antioxidants and the risk of OP using Mendelian randomization (MR).

**Methods:**

Published studies were used to identify instrumental variables related to absolute levels of circulating antioxidants like lycopene, retinol, ascorbate, and β-carotene, as well as antioxidant metabolites such as ascorbate, retinol, α-tocopherol, and γ-tocopherol. Outcome variables included BMD (in femoral neck, lumbar spine, forearm, heel, total body, total body (age over 60), total body (age 45–60), total body (age 30–45), total body (age 15–30), and total body (age 0–15)), fractures (in arm, spine, leg, heel, and osteoporotic fractures), and OP. Inverse variance weighted or Wald ratio was chosen as the main method for MR analysis based on the number of single nucleotide polymorphisms (SNPs). Furthermore, we performed sensitivity analyses to confirm the reliability of the findings.

**Results:**

We found a causal relationship between absolute retinol levels and heel BMD (*p* = 7.6E-05). The results of fixed effects IVW showed a protective effect of absolute retinol levels against heel BMD, with per 0.1 ln-transformed retinol being associated with a 28% increase in heel BMD (OR: 1.28, 95% CI: 1.13–1.44). In addition, a sex-specific effect of the absolute circulating retinol levels on the heel BMD has been observed in men. No other significant causal relationship was found.

**Conclusion:**

There is a positive causal relationship between absolute retinol levels and heel BMD. The implications of our results should be taken into account in future studies and in the creation of public health policies and OP prevention tactics.

## 1 Introduction

Osteoporosis (OP), recognized as the most prevalent systemic bone disorder, is primarily identified by reduced bone mineral density (BMD) and an increased incidence of brittle fractures resulting from the weakening of bone microarchitecture (Song et al., 2022). This deterioration can escalate the risk of severe disability or mortality, especially among older adults (Borgström et al., 2020). The universally accepted diagnostic approach for OP involves measuring BMD utilizing dual-energy x-ray absorptiometry, a technique that assesses BMD at specific skeletal sites including the femoral neck, lumbar spine, and forearm, which are particularly susceptible to changes in BMD and overall bone health (Kanis, 1997; Kanis, 2002). Additionally, in more recent times, the heel site has emerged as an additional location for estimating OP, further expanding the scope of diagnostic evaluation (Bai et al., 2020). Moreover, measuring total body BMD (TB-BMD) is also considered an equally valid and unbiased approach for evaluating overall BMD in the context of OP diagnosis. Fractures, another significant manifestation of OP, occur most commonly in the leg, arm, heel, and spine, further highlighting the widespread impact of the disease on skeletal integrity (Liu et al., 2019; Black et al., 2020; Leder et al., 2020). According to the most recent findings published by the International Osteoporosis Foundation, it is estimated that globally, one in three women and one in five men who are above the age of 50 are likely to suffer from OP (Cui et al., 2018; Liu et al., 2019; Black et al., 2020; Leder et al., 2020). This statistic not only reflects the extensive prevalence of this disease but also emphasizes the profound effect OP has on the quality of life of patients, as well as the substantial challenge it poses to public health infrastructures and national economies.

Oxidative stress occurs when there is an imbalance between oxidative and antioxidative processes in the body, a condition that significantly contributes to aging and various diseases (Mladenov et al., 2023). This imbalance is largely regulated by reactive oxygen species (ROS) and various antioxidant enzymes. Increasingly, studies are showing that bone health is largely influenced by the regulation of redox balance, with the control of ROS production in bone cells emerging as a critical strategy for preventing bone deterioration (Kaur et al., 2021). The activation of osteoclasts by receptor activator of NF-κB ligand (RANKL) through binding to RANK receptors on the surface of osteoclasts induces the formation of multinucleated giant cells, subsequently facilitating bone resorption and remodeling (Liang et al., 2023). The interaction between RANKL and ROS plays a key role in osteoclast activation and bone remodeling, forming a positive feedback loop to influence bone metabolic homeostasis (Tanaka et al., 2023; Xu et al., 2023). While the superoxide produced by osteoclasts plays a direct role in bone degradation, osteoblasts synthesize antioxidants like glutathione peroxidase (GPX) to combat ROS (Yuan et al., 2022). As a result, in scenarios where there is an imbalance between ROS production and antioxidant defense, it can adversely impact bone metabolism, potentially leading to OP (López-Armada et al., 2022). In addition, recent studies have shown that oxidative stress is involved in the pathogenesis of OP by uncoupling osteoclast and osteoblast functions (Baek et al., 2010; Manolagas, 2018). Using pathways of bone metabolism, such uncoupling can be achieved by promoting osteoclastogenesis, inducing apoptosis of osteoblasts and osteocytes, and inactivating osteoblasts (Kimball et al., 2021). Dietary antioxidants are commonly recognized as the most readily available source of antioxidant defense. Therefore, antioxidants, especially those from dietary sources, have an important role to play in the prevention of OP. There has been some research examining the relationship between certain dietary antioxidants and bone health in humans in the past (Sugiura et al., 2011; Talaulikar et al., 2012). Consuming more fruits, vegetables, and cereals reduces fracture risk, according to Warensjö et al. in a study of 56,736 women (Warensjö Lemming et al., 2017). Similar conclusions have been reached in other studies: natural antioxidant-rich plant foods can improve BMD (Platt et al., 2011; Wallace, 2017). Although diet-based supplements are easily accessible and affordable, it remains critical to identify specific circulating antioxidants that are causally associated with reduced risk of OP.

Randomized controlled trials (RCTs) are widely regarded as the most reliable method for determining causal relationships (Tew et al., 2023). However, RCTs may not always be feasible due to their potential high cost and ethical concerns. Additionally, the dosage and duration of antioxidant supplementations may limit the conclusions drawn from RCTs. As we know, it seems unrealistic to expect a few years of antioxidant treatment to reverse the effects of decades of oxidative stress. To address these limitations, an alternative statistical approach to explore causality in exposure-outcome relationships involves the utilization of instrumental variables. Specifically, Mendelian randomization (MR) is a powerful analytical method that uses genetic variation to study the causal impacts of risk factors on traits (Lawlor et al., 2008; Evans and Davey Smith, 2015; Burgess et al., 2019). Previous research using MR analysis has suggested possible causal links between levels of circulating antioxidants and a range of health conditions (Ni et al., 2023; Tang et al., 2023; Zhu et al., 2023; Chen et al., 2024; Zou et al., 2024). The primary objective of this study was to investigate the causal connection between diet-derived circulating antioxidants and the risk of OP.

## 2 Materials and methods

### 2.1 Study design

**FIGURE 1:**
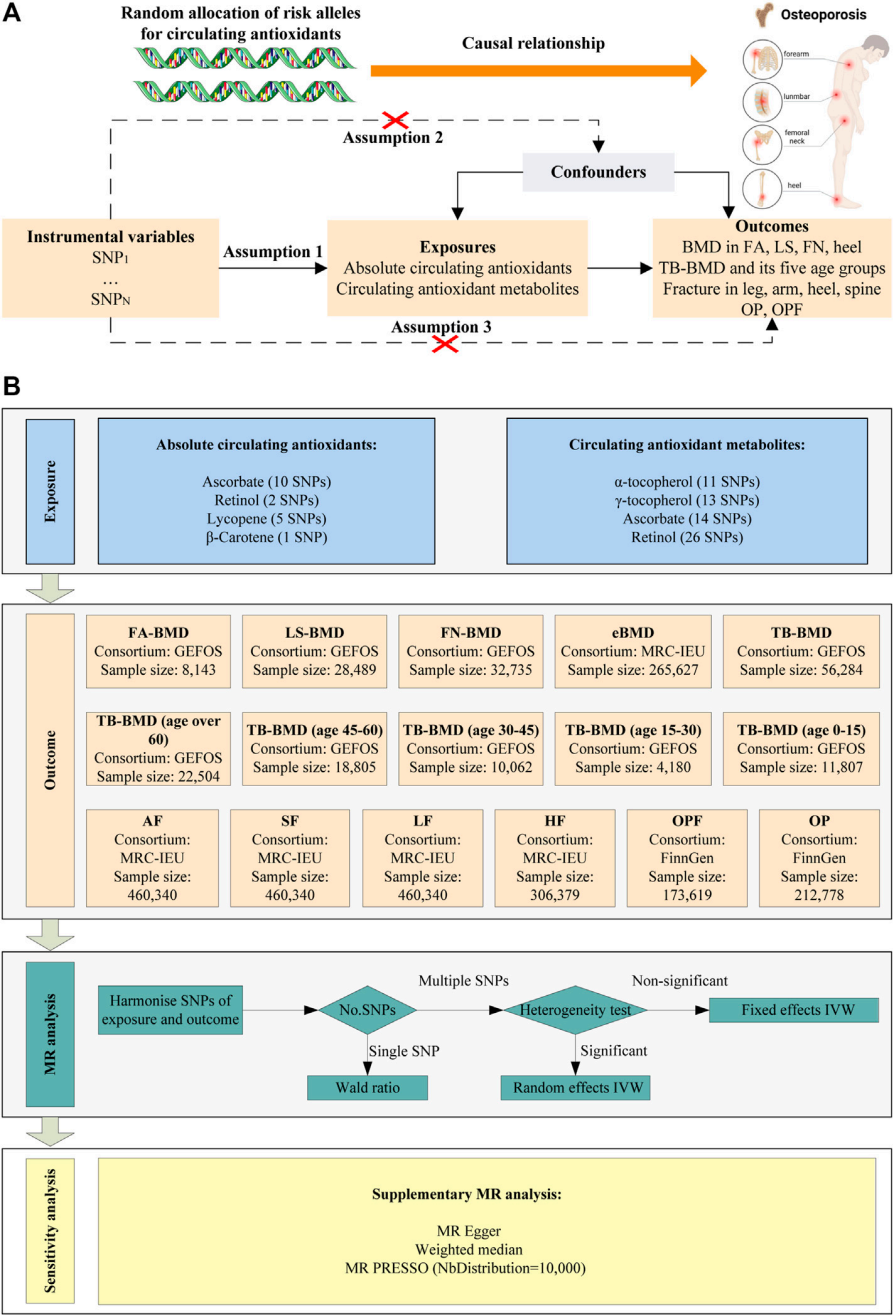
Schematic overview of the MR study design. **(A)** The three main assumptions of MR analysis. **(B)** Schematic overview and framework of the present MR study design.

The present study explored the causal relationship between circulating antioxidants and the risk of BMD, fractures, and OP using MR analysis based on Genome-wide association study (GWAS) summary data. For this purpose, we obtained two independent data sets regarding antioxidants: absolute circulating antioxidants and circulating antioxidant metabolites. Here, “absolute circulating antioxidants” refer to the genuine absolute blood levels, while “circulating antioxidant metabolites” denote relative concentrations. To ensure robustness in our MR analysis, it was imperative that all instrumental variables related to circulating antioxidants conform to three primary assumptions (Figure 1A). The overall design of this study was presented in Figure 1B. The data used in this study were obtained from publicly available databases, so ethical approval was not required.

### 2.2 Genetic instrumental variables selection

For absolute circulating ascorbate, retinol, and β-carotene, we set the following criteria to screen instrumental variables: *p* < 5 × 10^−8^, r^2^ < 0.001, and kb = 10,000. However, no SNP for lycopene was filtered based on the criterion of *p* < 5 × 10^−8^. Therefore, *p* < 5 × 10^−6^, r^2^ < 0.001, and kb = 10,000 were used as screening criteria. Overall, we screened 11 SNPs significantly associated with ascorbate from a recently published GWAS summary data involving 52,018 individuals (Zheng et al., 2021). After further analysis, the rs7740812 was excluded from the list of ascorbate-related SNPs due to linkage disequilibrium (LD) clumping. In a meta-analysis of GWAS involving 5,006 participants, we identified two independent SNPs associated with retinol (Mondul et al., 2011). In a GWAS from the Nurses’ Health Study, which included 2,344 participants, we identified two significantly associated SNPs for β-carotene (Hendrickson et al., 2012). One of the two identified SNPs, rs12934922, was later removed after LD clumping. In a GWAS involving 441 older Amish adults, we identified five independent SNPs associated with lycopene (D Adamo et al., 2016). Information on the GWAS cohort is provided in Supplementary Table S1.

For antioxidant metabolites, due to the lack of sufficient instrumental variables to perform MR analyses using strict screening criteria, we used looser thresholds (*p* < 1 × 10^−5^, r^2^ < 0.001, and kb = 10,000). The threshold has also recently been used to explore the causal relationship between antioxidants and osteoarthritis (Tang et al., 2023), inflammatory bowel disease (Zou et al., 2024), and digestive system tumors (Zhang et al., 2022). In brief, a GWAS with 7,824 participants identified independent SNPs linked to blood metabolites of α-tocopherol (n = 11), γ-tocopherol (n = 13), and ascorbate (n = 14) (Shin et al., 2014). For retinol, we extracted 26 SNPs as instrumental variables from a GWAS involving 1960 participants (Long et al., 2017).

According to previous reports, body mass index (BMI), alcohol consumption, and physical activity levels may be risk factors for OP (Kanis et al., 2019; Arceo-Mendoza and Camacho, 2021). To avoid the influence of confounders on the results, we obtained the largest GWAS to date on BMI, alcoholic drinks per week, and moderate-to-vigorous physical activity (Table 1). If antioxidant-related SNPs were also significant in the confounders, these SNPs were eliminated. To assess the strength of the instrumental variables, the *F*-statistic was also calculated (Palmer et al., 2012).

**TABLE 1:** Details for selected GWAS of confounders.

Confounders	Sample size	PMID
body mass index	681,275	30,124,842
Alcoholic drinks per week	335,394	30,643,251
Moderate to vigorous physical activity levels	377,234	29,899,525

### 2.3 Outcome data sources

It is well known that BMD loss in the forearm (FA-BMD), femoral neck (FN-BMD), lumbar spine (LS-BMD), and heel (eBMD) significantly increases the risk of OP and fractures more than in other parts of the body. Therefore, this study focuses on analyzing the causal relationship between circulating antioxidants and BMD in these four critical body parts. The GWAS summary data for FA-BMD, FN-BMD, and LS-BMD were obtained from the Genetic Factors for Osteoporosis Consortium (GEFOS) (Zheng et al., 2015), with participant numbers being 8,143, 32,735, and 28,498 respectively. Summary data for eBMD were derived from the United Kingdom Biobank (Wu et al., 2019), encompassing a total of 265,627 participants. The methodologies for measuring BMD have been elaborated in the original publication. Age is widely recognized as a risk factor for OP (Hadji et al., 2019). Hence, a comprehensive GWAS meta-analysis was employed to acquire detailed statistics on total body BMD (TB-BMD) across five age groups (under 15, 15–30, 30–45, 45–60, and above 60 years) (Medina-Gomez et al., 2018), involving 66,628 participants in total. OP leading to fractures is one of the most prevalent clinical symptoms (Wright et al., 2017). Therefore, GWAS summary data for fractures of the arm (AF; number of cases = 4,714, number of controls = 455,626), spine (SF; number of cases = 1,036, number of controls = 459,304), leg (LF; number of cases = 2,988, number of controls = 457,352), and heel (HF; sample size = 306,379) were obtained from the MRC-IEU database (Hemani et al., 2018). Furthermore, GWAS summary data for osteoporosis-related fractures (OPF; number of cases = 785, number of controls = 172,834) and OP (number of cases = 3,203, number of controls = 209,575) were downloaded from the FinnGen study (Kurki et al., 2023). The detailed data information for outcome is available in Table 2.

**TABLE 2:** The detailed data information for outcomes.

Outcome	Consortium	Population	Sample size	Sample case	Number of SNPs
FA-BMD	GEFOS	European	8,143	NA	9,955,366
LS-BMD	GEFOS	European	28,498	NA	10,582,867
FN-BMD	GEFOS	European	32,735	NA	10,586,900
eBMD	MRC-IEU	European	265,627	NA	9,851,867
TB-BMD	GEFOS	European	56,284	NA	16,162,733
TB-BMD (age over 60)	GEFOS	European	22,504	NA	11,932,096
TB-BMD (age 45–60)	GEFOS	European	18,805	NA	10,304,110
TB-BMD (age 30–45)	GEFOS	European	10,062	NA	9,656,698
TB-BMD (age 15–30)	GEFOS	European	4,180	NA	8,509,502
TB-BMD (age 0–15)	GEFOS	European	11,807	NA	9,351,693
AF	MRC-IEU	European	460,340	455,626	9,851,867
SF	MRC-IEU	European	460,340	459,304	9,851,867
LF	MRC-IEU	European	460,340	457,352	9,851,867
HF	MRC-IEU	European	306,379	NA	9,851,867
OPF	FinnGen	European	173,619	172,834	16,380,281
OP	FinnGen	European	212,778	209,575	16,380,452

FA-BMD, forearm bone mineral density; LS-BMD, lumbar spine bone mineral density; FN-BMD, femur neck bone mineral density; eBMD, heel bone mineral density; TB-BMD, total body bone mineral density; AF, arm fractures; SF, spine fractures; LF, leg fractures; HF, heel fractures; OPF, osteoporosis fracture; OP, osteoporosis; GEFOS, the Genetic Factors for osteoporosis Consortium; MRC-IEU, the Medical Research Council Integrative Epidemiology Unit.

### 2.4 Statistical analysis

Figure 1B displays the main approach used for MR analysis. First, the major effector allele of the SNPs was used to harmonize the exposure and outcome information. We removed SNPs that were inconsistent for effector alleles between exposure and outcome data. If only one SNP was ultimately included, we used the Wald ratio for MR estimates, owing to its efficacy in providing accurate estimations in such contexts (Boehm and Zhou, 2022). When more than one SNP was available, inverse variance weighting (IVW) was used as main method. However, in scenarios where the data encompassed multiple SNPs, we utilized Cochran’s Q test, a robust statistical tool, to evaluate the extent of heterogeneity among the SNPs. In instances where heterogeneity was identified, we adopted the random effects IVW model; conversely, if there was no evidence of heterogeneity, the fixed effects IVW model was our method of choice. The random effects IVW model is a commonly used method in MR analysis that accounts for the presence of heterogeneity by introducing random effects. Compared to fixed effects models, the random effects IVW model is more suitable for handling heterogeneity as it allows for differences between studies and assigns different weights to each study, thereby providing a more accurate estimation of the overall effect. According to the MR guidelines, IVW is deemed more effective than alternative methods in specific situations (Au Yeung and Gill, 2023). IVW, on the other hand, assumes that each genetic variant is a reliable tool, which may not always be the case in reality. Therefore, weighted median and MR Egger were used as supplementary methods to enhance the assessment of IVW. The MR Egger approach permits pleiotropic effects in all genetic variations, demanding that such effects are independent from the variant-exposure correlation. When less than half of the instrumental variables used in MR analysis are effective, the weighted median method permits the inclusion of ineffective instruments in evaluating causal effects. Finally, MR-PRESSO method was implemented for the identification and correction of outliers, ensuring the accuracy and reliability of our results.

In our primary MR analysis, a thorough evaluation was undertaken, which included an extensive examination of sixteen different outcomes, thereby providing a multifaceted perspective on the data under investigation. We employed the Bonferroni correction to address the issue of multiple testing, ensuring a more stringent and reliable determination of statistical significance. The Bonferroni correction is a commonly used method to control the error rate associated with multiple comparisons and reduce the risk of false positives. Specifically, we chose a threshold of *p* < 0.003 as the significance threshold. This threshold was determined by dividing 0.05 by the total number of outcomes. The *p*-values lower than 0.05 but not meeting the threshold for multiple-testing significance are considered as suggestive evidence of correlation. R software version 4.2.2 was used to perform the MR analysis using the TwoSampleMR (version 0.5.6) package.

## 3 Results

### 3.1 Selection of instrumental variables

Detailed information about the instrumental variables is presented in Table 3. The range of *R* ^2^ for circulating absolute antioxidants was 1.7%–30.1% (all *F*-statistics > 10), and the range of *R* ^2^ for circulating antioxidant metabolites was 6.8%–21.7% (all *F*-statistics > 10). All SNPs for instrumental variables are listed in Supplementary Tables S2 and S3. None of the included SNPs were significantly correlated with confounders (Supplementary Table S4).

**TABLE 3:** The summary of instrumental variables for circulating antioxidants.

Trait	Sample size	*p*	LD	No.SNPs	Explained variance (R2, %)	Unit	Population	PMID
Absolute circulating antioxidants								
Ascorbate	52,018	5 × 10^−8^	0.001	10	1.7	µmol/L	European	33,203,707
Lycopene	441	5 × 10^−6^	0.001	5	30.1	µg/dL	Amish	26,861,389
Retinol	5,006	5 × 10^−8^	0.001	2	2.3	µg/L in ln-transformed scale	Caucasian	21,878,437
β-Carotene	2,344	5 × 10^−8^	0.001	1	4.8	µg/L in ln-transformed scale	European	23,134,893
Circulating antioxidant metabolites								
α-Tocopherol	7,725	1 × 10^−5^	0.001	11	6.8	log10-transformed metabolites concentration	European	24,816,252
γ-Tocopherol	6,226	1 × 10^−5^	0.001	13	9.8	log10-transformed metabolites concentration	European	24,816,252
Ascorbate	2085	1 × 10^−5^	0.001	14	21.7	log10-transformed metabolites concentration	European	24,816,252
Retinol	1960	1 × 10^−5^	0.001	26	20.6	log10-transformed metabolites concentration	European	28,263,315

### 3.2 Absolute circulating antioxidants and osteoporosis

**FIGURE 2:**
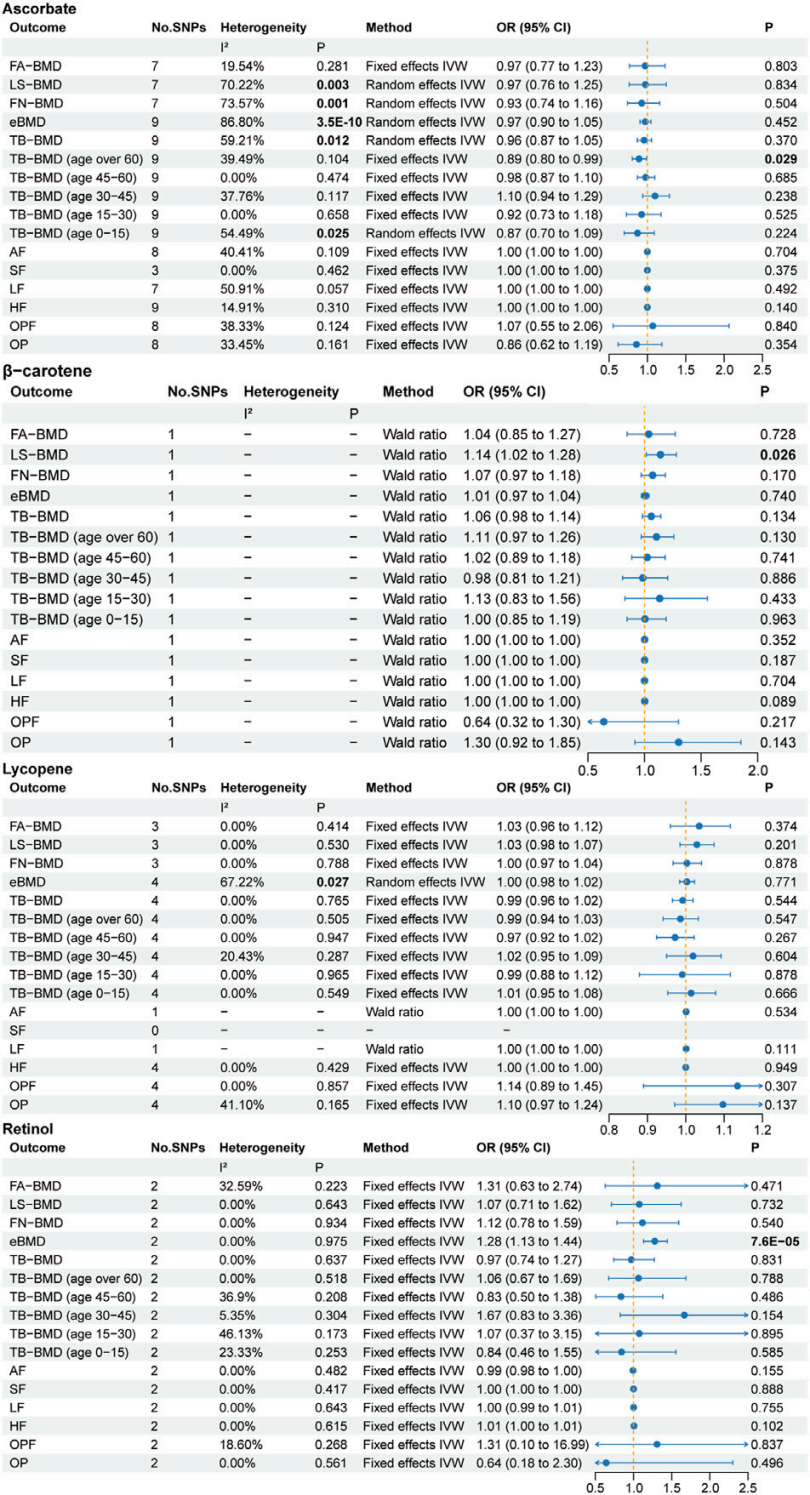
The primary MR analyses results of the causal effects of absolute circulating antioxidant levels on osteoporosis. Results with *p* less than 0.05 highlighted in bold. “-” represents not applicable.

**FIGURE 3:**
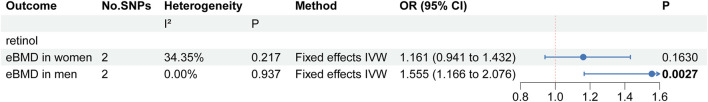
The MR analyses results of subgroup analyses. Results with *p* less than 0.05 highlighted in bold.

The primary MR results concerning absolute antioxidants are illustrated in the forest plot of Figure 2. Cochran’s Q statistic results suggested the presence of six heterogeneities, ascorbate in LS-BMD, FN-BMD, eBMD, TB-BMD and TB-BMD (age 0–15) and lycopene in eBMD. Heterogeneity may arise due to methodological differences between studies, differences in population characteristics, variations in the effects of genetic variants, among other factors. To enhance statistical power, we employed the random effects IVW method as the primary MR analysis approach. Based on the primary MR analysis method, we identified three causal relationships, including two suggestive associations and one significant association. We provide strong evidence of a positive correlation between retinol levels and eBMD (*p* = 7.6E-05), with a unit change in retinol levels leading to a 28% increase in eBMD (OR: 1.28, 95% CI: 1.13–1.44). Given the significant genetic influence on eBMD (Kim, 2018) and the potential impact of sex on the physiological effects of circulating antioxidants, we respectively downloaded the male and female sections of the eBMD GWAS data for further analysis. In a subgroup analysis of eBMD by sex, we found that circulating levels of retinol were significantly associated with increased eBMD in men, but not in women (Figure 3). Moreover, we found a negative correlation between ascorbate levels and TB-BMD (age over 60) (*p* = 0.029) and a positive correlation between β-carotene levels and LS-BMD (*p* = 0.026), but it should be noted that these evidences are quite weak. Regarding the other traits, no potential causal links were observed.

For ascorbate and lycopene, the *p*-values of the MR Egger intercept test ranged from 0.095 to 0.978, indicating no pleiotropy (Supplementary Table S5). Due to the limited number of SNPs available, sensitivity analyses for β-carotene and retinol could not be conducted. In addition, utilizing supplementary MR analysis methods, we found no association between ascorbate levels and TB-BMD (age over 60) (Supplementary Table S6). Interestingly, the weighted median results revealed a significant negative correlation between ascorbate levels and eBMD (*p* = 0.001). However, after the elimination of outliers using the MR PRESSO method, there was no significant association found between the two (*p* = 0.471), suggesting potential bias caused by outliers.

### 3.3 Circulating antioxidant metabolites and osteoporosis

**FIGURE 4:**
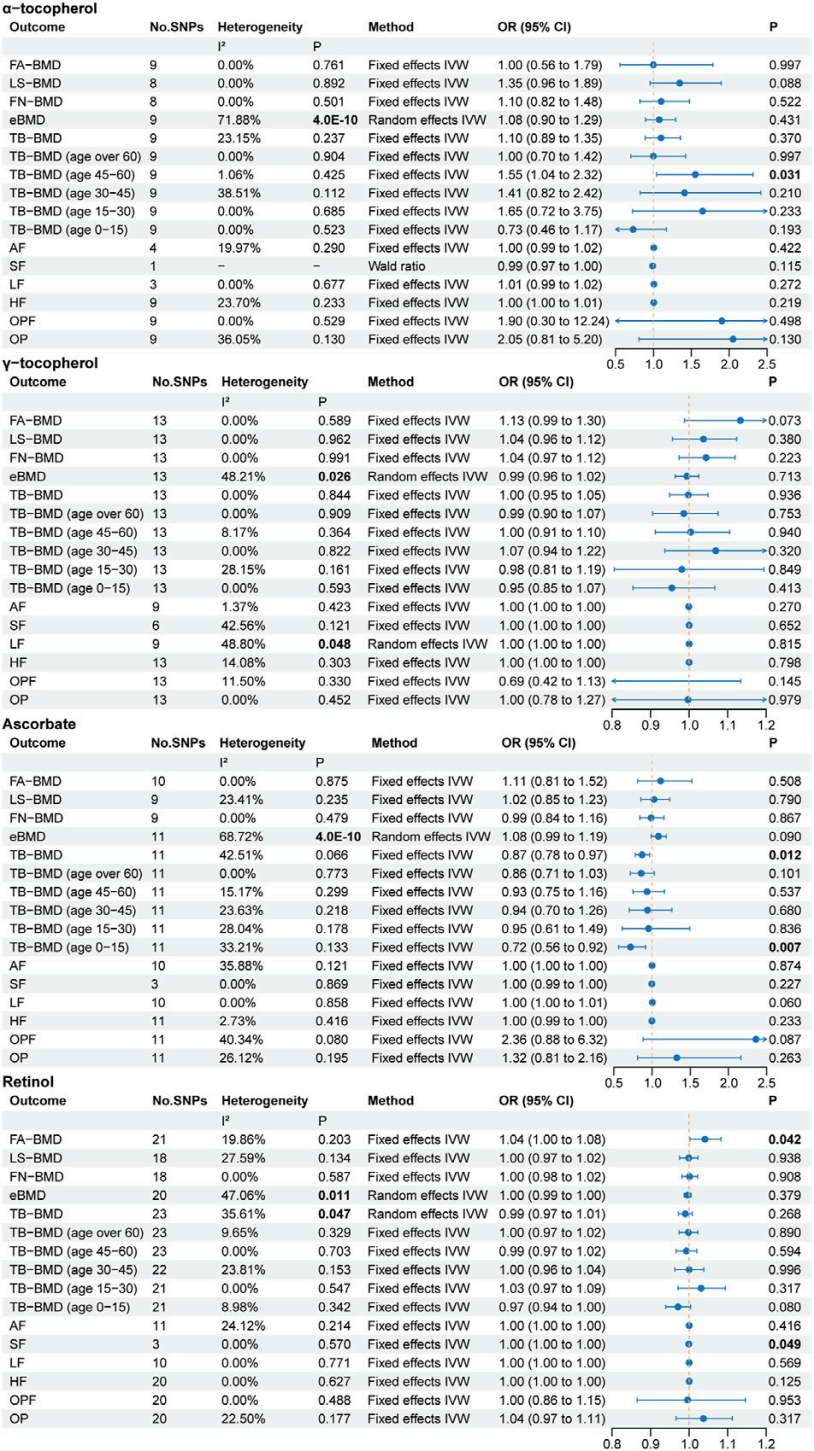
The primary MR analyses results of the causal effects of circulating antioxidant metabolites on osteoporosis. Results with *p* less than 0.05 highlighted in bold. “-” represents not applicable.

The primary MR results regarding antioxidant metabolites are depicted in the forest plot of Figure 4. Cochran’s Q statistic results also suggested the presence of five heterogeneities, α-tocopherol in eBMD, γ-tocopherol in eBMD and LF, ascorbate in eBMD, and retinol in eBMD and TB-BMD. In the MR analysis of circulating antioxidant metabolites, we did not find evidence of significant associations. However, we identified five suggestive correlation evidences: α-tocopherol levels positively correlated with TB-BMD (age 45–60), ascorbate levels negatively correlated with TB-BMD and TB-BMD (age 0–15), retinol levels positively correlated with FA-BMD, and negatively with SF (OR = 0.999). As earlier mentioned, these pieces of evidence are weak.

In the MR Egger intercept test for circulating antioxidant metabolites, we found potential pleiotropy for α-tocopherol in TB-BMD (*p* = 0.049) (Supplementary Table S7). However, the MR PRESSO did not identify any outliers affecting this causal relationship assessment (Supplementary Table S8). In the supplementary MR analysis, there was no evidence to further support the suggestive associations. In the assessment of the causal relationship between four types of circulating antioxidant metabolites and eBMD, MR PRESSO identified outliers in each case. However, the MR analysis results after outlier removal were consistent with the primary MR analysis results.

## 4 Discussion

With the significant increase in the incidence of OP over the past few decades, it has become increasingly important to recognize modifiable risk factors, particularly dietary factors, as a promising strategy to curb the occurrence and progression of these diseases. Building on this understanding, in this study, we used genetic variations in the concentration of antioxidants as instrumental variables for exposure. Expanding the scope of our research, our outcome data encompasses not just OP and OPF, but also BMD across different body areas and age groups, along with fractures in various body locations. In terms of specific findings, our results demonstrate a notable positive association between absolute levels of retinol and eBMD. Interestingly, we also found that this significant causal relationship was related to gender. Additionally, we identified weak causal relationships of absolute ascorbate in TB-BMD (age over 60), β-carotene in LS-BMD, α-tocopherol in TB-BMD (age 45–60), relative ascorbate in TB-BMD and TB-BMD (age 0–15), and relative retinol in FA-BMD and SF. However, there was no evidence to support a causal relationship between other circulating antioxidants and the outcomes.

The relationships between antioxidants and the risk of OP have been evaluated in a number of observational studies. Yet, the question of whether antioxidants can mitigate the risk of OP remains debated. While some epidemiological research indicates that high levels of vitamin A (retinol) intake or serum vitamin A are linked to a negative impact on bone mass, this is not a universally accepted finding. Other studies contradict these findings, with some researchers suggesting that vitamin A may actually enhance bone health. For instance, an epidemiological study from Norway demonstrated that elevated serum vitamin A levels did not lead to a higher incidence of hip fractures (Holvik et al., 2015). In contrast, a meta-analysis by Wu et al. (Wu et al., 2014), encompassing eight studies on vitamin A intake, revealed a correlation between high vitamin A consumption and an increased risk of hip fractures. Similarly, a meta-analysis by Zhang et al. (Zhang et al., 2017a), involving 13 studies, showed that increased vitamin A intake was linked to a lower risk of fractures at various sites, but with a notable increase in the risk of hip fractures. However, another piece of research reported an increase in BMD levels with higher vitamin A intake, leading to conflicting conclusions (Barker et al., 2005). In the context of our MR study, absolute retinol levels were significantly positively correlated with eBMD in men. In addition, there were suggestive associations found between metabolic levels of retinol and increased FA-BMD (OR = 1.041, *p* = 0.042) and decreased risk of SF (OR = 0.999, *p* = 0.049). Those with OP may benefit from a diet rich in antioxidants, especially vitamin A, and an antioxidant-preserving lifestyle. Vitamin A exerts its effects through the metabolite all-trans-retinoic acid (ATRA), a potent transcriptional regulator that modulates the expression of genes crucial for mediating the indirect antioxidant properties of vitamin A. Consequently, vitamin A can activate the NRF2/KEAP1 or NF-κB signaling pathways to mitigate oxidative stress and decrease the risk of OP (Dinkova-Kostova and Talalay, 2008; Abraham et al., 2019). However, larger clinical trials are needed to increase the credibility of clinical recommendations, especially given the presence of weak causality and sex-specific effects of retinol.

As a key antioxidant, Vitamin C (ascorbate) plays a crucial role in removing ROS, thereby reducing oxidative stress (Kim and Lee, 2016). Vitamin C has been shown to stimulate the formation of osteoclasts and osteoblasts *in vitro*, although high doses of the vitamin can also exhibit cytotoxic effects on both cell types (Chin and Ima-Nirwana, 2018). New et al. discovered a non-linear relationship between vitamin C consumption and BMD in a cohort of 994 healthy premenopausal women aged 45–49 participating in the Osteoporosis Screening Program in Aberdeen (New et al., 1997). According to a report conducted by Kim and Lee (Kim and Lee, 2016), an elevated intake of vitamin C may decrease the likelihood of OP among Korean adults over 50 who have low levels of physical activity, but surprisingly, this benefit does not extend to those with higher levels of physical activity. Moreover, increased dietary intake of vitamin C has been linked to a decreased risk of hip fractures and OP, as well as to enhanced BMD, notably in the femoral neck and lumbar spine areas (Malmir et al., 2018). Simon and colleagues discovered a significant nonlinear relationship between serum vitamin C levels and hip BMD in men (Simon and Hudes, 2001). Specifically, hip BMD initially increased with rising serum vitamin C levels, but subsequently decreased as serum vitamin C levels continued to rise. Interestingly, while primarily known for its antioxidant effects, vitamin C can display prooxidant characteristics at high doses, as indicated by a previous study where a daily supplementation of 500 mg of vitamin C in individuals aged 17 to 49 for 6 weeks led to oxidative DNA damage (Podmore et al., 1998), a factor potentially relevant to OP (Finck et al., 2014). The current study identified three suggestive causal associations between absolute ascorbate concentrations and reduced TB-BMD (age over 60) and metabolic concentrations of ascorbate with reduced TB-BMD and TB-BMD (age 0–15). However, higher-quality trials are necessary to determine if Vitamin C significantly reduces the risk of OP.

Vitamin E, known for its antioxidant properties, is divided into two primary subgroups, namely, tocopherols and tocotrienols. Each of these subgroups contains four unique analogs: alpha, beta, gamma, and delta (Zhang et al., 2017b). Significantly, α-tocopherol is one of the homologs within the vitamin E category. Research suggests that vitamin E may benefit bone health due to its anti-inflammatory properties, yet this effect has not been conclusively demonstrated in human subjects (Reid and New, 1997). To further explore this, Shi et al. (Shi et al., 2016) investigated the correlation between vitamin E levels and BMD in middle-aged and elderly adults (40–75 years) in Guangzhou, China. Their findings indicated a positive association between higher intake and serum levels of vitamin E and increased BMD in Chinese women, but this association was not mirrored in Chinese men. These results are similar to our findings, as our MR analysis revealed a suggestive association between the metabolic level of α-tocopherol and increased TB-BMD in individuals aged 45–60. In contrast, a negative correlation has been observed between serum α-tocopherol levels and femoral neck bone mineral density among the elderly population in the US (both men and women over 50), indicating a potentially detrimental impact of α-tocopherol on bone health (Zhang et al., 2017b). γ-tocopherol, the second most prevalent isoform of vitamin E found in dietary sources, accounts for 20%–25% of total vitamin E intake and exhibits the ability to scavenge both reactive nitrogen species (RNS) and ROS, distinguishing it from α-tocopherol (Abraham et al., 2019). While a clinical trial indicated that supplementation with γ-tocopherol reduces systemic oxidative stress, findings from cross-sectional studies have been inconclusive. Yang et al. (Yang et al., 2016) did not observe statistically significant associations between γ-tocopherol and BMD in a study involving over 5,000 older women. In contrast, Ilesanmi-Oyelere et al. (Ilesanmi-Oyelere et al., 2019) reported that increased vitamin E intake was linked to lower BMD levels. The current body of evidence regarding the impact of γ-tocopherol on OP remains limited, highlighting the need for more rigorous trials to elucidate its effects.

Lycopene is an acyclic isomer derived from β-carotene (Agarwal and Rao, 2000). A long-term study over 17 years found that increased lycopene consumption was associated with a lower incidence of hip and nonvertebral fractures, highlighting its potential role in bone health (Sahni et al., 2009). This elevation in lycopene potentially lowers oxidative stress and bone resorption markers, thereby suggesting a possible reduction in the risk of OP. Nevertheless, a cross-sectional study involving 4,820 individuals in the United States revealed no significant association between lycopene intake and OP (Kan et al., 2021). There is weak evidence that β-carotene levels are associated with increased LS-BMD in the current study. However, the results of a previous MR performed by li et al. (Li et al., 2023) showed that serum β-carotene levels significantly reduced OP risk. For the instrumental variables of β-carotene, we used more strict screening criteria (*p* < 5 × 10^−8^ and r^2^ < 0.001), which helped us to make more reliable conclusions. Furthermore, in our study, we also analyzed subgroups of TB-BMD according to age. In conclusion, the weak causal relationship between β-carotene levels and increased LS-BMD provides some insight into the reduction of OP risk by dietary antioxidants.

The study we conducted possesses a range of strengths. Primarily, the MR approach, utilizing two separate samples, reduces the potential risks that participants may encounter in clinical trials. Additionally, the application of distinct sets of instrumental variables for both absolute circulating antioxidants and their metabolites increases the informative value of the MR study outcomes. However, our study does have its limitations. One of the primary limitations is the constrained number of SNPs for antioxidant instrumental variables that were sourced from published GWAS data. In other MR analysis, absolute retinol and β-carotene were also unable to validate the robustness of the results using complementary MR analysis methods due to limitations in the number of SNPs (Mondul et al., 2011; Hendrickson et al., 2012; Zhao et al., 2023). It should be noted, however, that these SNPs are situated within essential genes pertinent to antioxidant metabolism, according to the PhenoScanner database (Kamat et al., 2019). In future studies, identifying more relevant loci through broader GWAS will be critical to strengthen the effectiveness of the instrumental variables. Another point is that antioxidant concentrations vary greatly with different detection methods, underscoring the need for standardizing these techniques in future studies. Finally, postmenopausal status was not included in the subgroup analyses due to data limitations, which may have weakened the reliability of our conclusions. Broader GWAS data are needed for more detailed subgroup analyses in the future.

## 5 Conclusion

In conclusion, there is a positive causal relationship between absolute retinol levels and eBMD. Our study, however, was unable to establish evidence of a causal relationship between retinol and BMD at other body sites or fractures. The implications of these results should be taken into account in future studies and in the creation of public health policies and OP prevention tactics.

## Data Availability

The original contributions presented in the study are included in the article/Supplementary Material, further inquiries can be directed to the corresponding author.
