# Supplementary material for: Elucidating causal relationships of diet-derived circulating antioxidants and the risk of osteoporosis: A Mendelian randomization study
Source: Front Genet. 2024 Jun 7;15:1346367. doi: 10.3389/fgene.2024.1346367 (PMC11190308; doi:10.3389/fgene.2024.1346367)
Supplement: Supplementary file 1 [file DataSheet1.PDF]

Supplementary Material

Elucidating causal relationships of diet-derived circulating antioxidants and the risk of osteoporosis: A Mendelian randomization study

Kexin Yuan[1†], Xingwen Xie[2†], Weiwei Huang[1], Dingpeng Li[3], Yongli Zhao[2], Haodong Yang[1], and Xuetao Wang[1*]

[1] Gansu University of Chinese Medicine, Lanzhou, Gansu 73000, China.

[2] Affiliated Hospital of Gansu University of Chinese Medicine, Lanzhou, Gansu 73000, China.

[3] The Second People's Hospital of Gansu Province, Lanzhou, Gansu 73030, China.

[†]These authors have contributed equally to this work

*** Correspondence:**
Xuetao Wang
18317521548@163.com

Supplementary Table 1 The summary information for circulating antioxidants.

Trait	Sample size	Age (years)	Sex (male, %)	Measurement method	Concentration
**Absolute circulating antioxidants**					
**Ascorbate (umol/L)**					
Fenland GWAS array	1,349	45±7.0	44%	Fluorometric assay	66.2±21.3
Fenland UKBB array	8,391	49±7.0	47%	Fluorometric assay	68.6±21.5
InterAct subcohort GWAS	3,521	51±9.0	35%	High-performance liquid chromatography with ultraviolet detection	42.8±19.0
InterAct subcohort core-exome	6,504	53±9.0	38%	High-performance liquid chromatography with ultraviolet detection	42.9±19.1
InterAct non-subcohort GWAS	2,944	55±8.0	48%	High-performance liquid chromatography with ultraviolet detection	36.4±17.6
InterAct non-subcohort core-exome	3,872	56±7.0	52%	High-performance liquid chromatography with ultraviolet detection	36.5±18.9
EPIC-Norfolk GWAS	16,756	59±9.0	47%	Fluorometric assay	53.8±20.2
EPIC-CVD subcohort	885	53±12.0	41%	High-performance liquid chromatography with ultraviolet detection	41.0±21.0

EPIC-CVD non-subcohort	6,765	57±8.0	55%	High-performance liquid chromatography with ultraviolet detection	37.9±20.8
**Lycopene (µg/dL)**					
HAPI	441	43.1±13.0	58%	Reverse-phase high-pressure liquid chromatography	39.2±19.9
**Retinol (µg/dL)**					
ATBC	4,014	58.1±5.0	100%	Reversed-phase liquid chromatography with diode-array UV detection	572 (796-654)
PLCO	992	64.6 ± 4.9	100%	Reversed-phase liquid chromatography with diode-array UV detection	672 (562-794)
**β-Carotene (µg/L)**					
NHS	2,344	58.8±6.4	0%	Reverse-phase high-pressure liquid chromatography	303±258
**Circulating antioxidant metabolites**					
**α-tocopherol**					
TwinsUK	5,966	53.4±14.0	7%	Liquid-phase chromatography and gas chromatography separation coupled with tandem mass spectrometry	NA
KORA	1,759	60.8±8.8	49%	Liquid-phase chromatography and gas chromatography separation coupled with tandem mass spectrometry	NA
**γ-tocopherol**					

TwinsUK	5,249	53.4±14.0	7%	Liquid-phase chromatography and gas chromatography separation coupled with tandem mass spectrometry	NA
KORA	977	60.8±8.8	49%	Liquid-phase chromatography and gas chromatography separation coupled with tandem mass spectrometry	NA
**Ascorbate**					
TwinsUK	518	53.4±14.0	7%	Liquid-phase chromatography and gas chromatography separation coupled with tandem mass spectrometry	NA
KORA	1,567	60.8±8.8	49%	Liquid-phase chromatography and gas chromatography separation coupled with tandem mass spectrometry	NA
**Retinol**					
TwinsUK	1,960	58 (32–87)	NA	The non-targeted metabolomics analysis was performed at Metabolon (Durham, North Carolina, USA) on a platform consisting of four independent ultra-high-performance liquid chromatography–tandem mass spectrometry instruments	NA

ATBC: Alpha-Tocopherol, Beta-Carotene Cancer Prevention Study; EPIC: European Prospective Investigation into Cancer and Nutrition; InCH: InCHIANTI Study; KORA: The Cooperative Health Research in the Region of Augsburg; NHS: Nurses' Health Study; PLCO: Prostate, Lung, Colorectal, and Ovarian (PLCO) Cancer Screening Trial; HAPI: Heredity and Phenotype Intervention Heart Study; NA, not applicable.

Supplementary Table 2 Instrumental variables associated with absolute circulating antioxidants.

Antioxidant	SNP	Effect allele	Other allele	EAF	*F*-statistic†	Beta	SE	*P*
Ascorbate	rs6693447	T	G	0.55	42.25	0.039	0.006	6.25E-10
	rs13028225	T	C	0.86	128.44	0.102	0.009	2.38E-30
	rs33972313	C	T	0.97	400.00	0.360	0.018	4.61E-90
	rs10051765	C	T	0.34	31.04	0.039	0.007	3.64E-09
	rs174547	C	T	0.33	26.45	0.036	0.007	3.84E-08
	rs117885456	A	G	0.09	42.25	0.078	0.012	1.70E-11
	rs2559850	A	G	0.60	93.44	0.058	0.006	6.30E-20
	rs10136000	A	G	0.28	32.65	0.040	0.007	1.33E-08
	rs56738967	C	G	0.32	34.31	0.041	0.007	7.62E-10
	rs9895661	T	C	0.82	62.02	0.063	0.008	1.05E-14
Retinol	rs10882272	C	T	0.35	56.25	-0.030	0.004	6.51E-15
	rs1667255	C	A	0.31	56.25	0.030	0.004	6.35E-14
Lycopene	rs7680948	A	T	0.20	40.11	-0.190	0.030	4.97E-09
	rs4635297	A	C	0.08	27.04	0.260	0.050	6.46E-07
	rs341075	A	G	0.02	26.19	-0.870	0.170	5.75E-07
	rs6108801	C	T	0.04	28.44	-0.480	0.090	4.07E-07
	rs2232315	A	G	0.03	24.34	0.740	0.150	1.26E-06
β-Carotene	rs6564851	G	T	0.36	98.67	0.149	0.015	1.60E-24

†The F-statistic for each SNP was calculated by the following formula: *F*-statistic = $Beta^2/SE^2$. EAF: effect allele frequency.

Supplementary Table 3 Instrumental variables associated with circulating antioxidant metabolites.

Antioxidant	SNP	Effect allele	Other allele	EAF	*F*-statistic†	Beta	SE	*P*
α-Tocopherol	rs10935814	A	G	0.10	19.87	-0.037	0.008	9.44E-06
	rs1404410	G	C	0.21	20.60	0.024	0.005	4.57E-06
	rs10245705	T	C	0.02	27.25	-0.066	0.013	1.95E-07
	rs11992435	G	A	0.05	20.63	-0.033	0.007	6.38E-06
	rs11145330	C	A	0.11	22.70	-0.032	0.007	1.95E-06
	rs7930821	T	C	0.02	19.98	0.067	0.015	7.53E-06
	rs261342	C	G	0.79	20.86	-0.017	0.004	5.41E-06
	rs1532701	A	G	0.55	20.85	0.014	0.003	5.07E-06
	rs10163969	T	G	0.04	19.36	-0.035	0.008	9.38E-06
	rs7238006	C	T	0.07	24.30	-0.028	0.006	6.77E-07
	rs2074731	A	C	0.17	22.26	-0.018	0.004	2.31E-06
γ-Tocopherol	rs6713914	C	T	0.43	26.13	-0.059	0.012	3.22E-07
	rs13069990	T	C	0.38	21.16	-0.051	0.011	4.44E-06
	rs6834631	G	T	0.04	23.93	-0.131	0.027	1.03E-06
	rs13103690	G	T	0.46	20.77	0.047	0.010	5.20E-06
	rs6826474	T	C	0.04	23.06	-0.138	0.029	1.56E-06
	rs2070006	C	T	0.63	20.90	-0.051	0.011	4.76E-06
	rs11167905	C	T	0.15	24.03	-0.080	0.016	9.83E-07
	rs9419004	C	G	0.19	20.35	-0.254	0.056	6.53E-06
	rs7112460	T	C	0.07	23.63	0.108	0.022	1.14E-06
	rs8057559	T	C	0.03	19.74	0.140	0.031	9.10E-06
	rs8105491	T	G	0.15	22.24	-0.070	0.015	2.30E-06
	rs808686	A	G	0.61	21.83	0.060	0.013	3.01E-06
	rs9606290	A	G	0.24	20.40	0.159	0.035	6.32E-06
	rs577596	A	G	0.33	24.74	-0.057	0.011	6.68E-07
Ascorbate	rs2794327	T	C	0.67	19.69	-0.036	0.008	8.78E-06
	rs6821770	A	G	0.14	19.52	0.038	0.009	8.92E-06

	rs10077932	T	C	0.14	21.35	-0.040	0.009	4.08E-06
	rs10520845	A	C	0.02	20.78	0.191	0.042	5.27E-06
	rs7038957	C	T	0.17	21.43	0.029	0.006	3.86E-06
	rs10492212	T	C	0.16	19.53	-0.027	0.006	8.66E-06
	rs10466757	T	A	0.84	19.66	-0.063	0.014	9.56E-06
	rs7350776	G	C	0.30	21.12	-0.024	0.005	3.86E-06
	rs261301	C	T	0.87	22.56	-0.032	0.007	2.06E-06
	rs13336771	A	C	0.17	20.15	0.062	0.014	7.39E-06
	rs1013104	T	C	0.44	21.16	-0.021	0.005	3.83E-06
	rs1060467	G	A	0.41	26.81	-0.023	0.005	2.61E-07
	rs5994305	G	A	0.17	24.52	-0.031	0.006	7.15E-07
Retinol	rs10019071	A	G	0.02	16.65	0.657	0.161	3.64E-06
	rs112293959	G	A	0.03	10.89	-0.429	0.130	5.70E-06
	rs114515641	G	T	0.03	10.30	0.414	0.129	7.12E-06
	rs1153379	A	G	0.93	15.14	-0.323	0.083	6.10E-06
	rs1176744	C	A	0.32	21.16	-0.207	0.045	3.50E-07
	rs118025446	A	G	0.03	17.49	-0.481	0.115	9.84E-06
	rs12955464	G	C	0.14	14.72	-0.234	0.061	3.71E-06
	rs139726207	G	A	0.04	11.31	0.370	0.110	4.46E-06
	rs149113848	G	C	0.01	13.23	-0.964	0.265	3.47E-06
	rs149478645	G	A	0.02	12.75	-0.507	0.142	1.30E-06
	rs17005512	C	G	0.17	13.20	-0.218	0.060	2.77E-06
	rs1842947	G	A	0.52	19.94	-0.192	0.043	8.34E-07
	rs2147337	G	T	0.66	12.89	0.158	0.044	9.01E-06
	rs2367816	G	A	0.77	19.99	0.228	0.051	9.46E-06
	rs2417325	T	C	0.93	15.34	0.329	0.084	1.29E-06
	rs3890033	C	T	0.38	11.76	0.144	0.042	8.56E-06
	rs3898702	T	C	0.20	16.15	-0.217	0.054	3.02E-06
	rs4135385	G	A	0.24	18.72	0.212	0.049	9.80E-06
	rs58411567	A	G	0.22	16.00	-0.208	0.052	3.02E-07
	rs6550239	A	G	0.74	14.54	-0.183	0.048	4.40E-06

rs75308833	T	C	0.02	11.29	-0.494	0.147	3.51E-06
rs7926028	T	G	0.45	10.33	-0.135	0.042	2.75E-06
rs945817	A	G	0.19	25.00	-0.275	0.055	6.46E-07
rs9586119	C	T	0.07	18.29	0.355	0.083	3.34E-06
rs117468033	T	C	0.01	26.13	-0.961	0.188	8.40E-06
rs568632536	T	C	0.03	14.39	0.531	0.140	8.08E-06

†The F-statistic for each SNP was calculated by the following formula: *F*-statistic = $Beta^2/SE^2$. EAF: effect allele frequency.

Supplementary Table 4 Instrumental variables associated with confounders.

SNP	Confounder	Pval.confounder
rs6564851	body mass index	0.580
rs4635297	body mass index	0.340
rs1667255	body mass index	0.910
rs2232315	body mass index	0.380
rs9895661	body mass index	0.150
rs7680948	body mass index	0.600
rs174547	body mass index	0.100
rs341075	body mass index	0.820
rs6108801	body mass index	0.650
rs341075	Alcoholic drinks per week	0.888
rs4635297	Alcoholic drinks per week	0.606
rs174547	Alcoholic drinks per week	0.004
rs7680948	Alcoholic drinks per week	0.225
rs6693447	Alcoholic drinks per week	0.364
rs1667255	Alcoholic drinks per week	0.599
rs9895661	Alcoholic drinks per week	0.202
rs6564851	Alcoholic drinks per week	0.414
rs10051765	Alcoholic drinks per week	0.225
rs2232315	Alcoholic drinks per week	0.136
rs2559850	Alcoholic drinks per week	0.935
rs10136000	Alcoholic drinks per week	0.483
rs6108801	Alcoholic drinks per week	0.067
rs33972313	Alcoholic drinks per week	0.017
rs56738967	Alcoholic drinks per week	0.061
rs117885456	Alcoholic drinks per week	0.724
rs6564851	Moderate to vigorous physical activity levels	0.094
rs117885456	Moderate to vigorous physical activity levels	0.660
rs10051765	Moderate to vigorous physical activity levels	0.400

rs6108801	Moderate to vigorous physical activity levels	0.820
rs33972313	Moderate to vigorous physical activity levels	0.470
rs174547	Moderate to vigorous physical activity levels	0.033
rs56738967	Moderate to vigorous physical activity levels	0.220
rs2232315	Moderate to vigorous physical activity levels	0.940
rs10882272	Moderate to vigorous physical activity levels	0.830
rs4635297	Moderate to vigorous physical activity levels	0.570
rs10136000	Moderate to vigorous physical activity levels	0.370
rs2559850	Moderate to vigorous physical activity levels	0.240
rs7680948	Moderate to vigorous physical activity levels	0.830
rs13028225	Moderate to vigorous physical activity levels	0.006
rs1667255	Moderate to vigorous physical activity levels	0.120
rs341075	Moderate to vigorous physical activity levels	0.170
rs6693447	Moderate to vigorous physical activity levels	0.370
rs9895661	Moderate to vigorous physical activity levels	0.210

Supplementary Table 5 The MR-Egger intercept test results for absolute circulating antioxidants.

**Outcome**	**Exposure**	**MR Egger intercept**	***P***
FA-BMD	Ascorbate	0.032	0.179
LS-BMD	Ascorbate	0.028	0.205
FN-BMD	Ascorbate	0.007	0.757
eBMD	Ascorbate	0.007	0.150
TB-BMD	Ascorbate	-0.005	0.386
TB-BMD (age over 60)	Ascorbate	-0.004	0.628
TB-BMD (age 45-60)	Ascorbate	0.005	0.516
TB-BMD (age 30-45)	Ascorbate	-0.018	0.150
TB-BMD (age 15-30)	Ascorbate	-0.007	0.659
TB-BMD (age 0-15)	Ascorbate	-0.016	0.237
AF	Ascorbate	0.000	0.181
SF	Ascorbate	0.000	0.498
LF	Ascorbate	0.000	0.518
HF	Ascorbate	0.000	0.978
OPF	Ascorbate	0.041	0.411
OP	Ascorbate	0.036	0.095
FA-BMD	Lycopene	0.044	0.412
LS-BMD	Lycopene	-0.020	0.481
FN-BMD	Lycopene	0.001	0.969
eBMD	Lycopene	0.005	0.681
TB-BMD	Lycopene	-0.010	0.498
TB-BMD (age over 60)	Lycopene	-0.025	0.365
TB-BMD (age 45-60)	Lycopene	0.009	0.734
TB-BMD (age 30-45)	Lycopene	-0.008	0.870
TB-BMD (age 15-30)	Lycopene	-0.023	0.708
TB-BMD (age 0-15)	Lycopene	-0.008	0.805
HF	Lycopene	0.000	0.939
OPF	Lycopene	0.036	0.779
OP	Lycopene	-0.106	0.198

Results with *P* less than 0.05 highlighted in bold. FA-BMD, forearm bone mineral density; LS-BMD, lumbar spine bone mineral density; FN-BMD, femur neck bone mineral density; eBMD, heel bone mineral density; TB-BMD, total body bone mineral density; AF, arm fractures; SF, spine fractures; LF, leg fractures; HF, heel fractures; OPF, osteoporosis fracture; OP, osteoporosis.

Supplementary Table 6 The complementary MR analyses results for absolute circulating antioxidants.

MR method	Ascorbate			Lycopene		
	No.SNPs*	OR (95% CI)	*P*	No.SNPs*	OR (95% CI)	*P*
**FA-BMD**						
MR Egger	7	0.579 (0.291 to 1.154)	0.181	3	0.952(0.824- 1.100)	0.626
Weighted median	7	0.883 (0.652 to 1.195)	0.420	3	1.004(0.917- 1.099)	0.928
MR PRESSO		NA	NA		NA	NA
**LS-BMD**						
MR Egger	7	0.615 (0.318 to 1.188)	0.207	3	1.068(0.984- 1.160)	0.361
Weighted median	7	0.844 (0.704 to 1.012)	0.067	3	1.032(0.977- 1.089)	0.258
MR PRESSO		0.918 (0.730 to 1.154)	0.464		NA	NA
**FN-BMD**						
MR Egger	7	0.829 (0.407 to 1.686)	0.626	3	1.001(0.933- 1.074)	0.975
Weighted median	7	0.934 (0.787 to 1.109)	0.438	3	1.008(0.968- 1.050)	0.698
MR PRESSO		0.931 (0.779 to 1.111)	0.428		NA	NA
**eBMD**						
MR Egger	9	0.911 (0.821 to 1.010)	0.120	4	0.993(0.947- 1.041)	0.796
Weighted median	9	0.945 (0.913 to 0.978)	**0.001**	4	1.003(0.985- 1.021)	0.761
MR PRESSO		0.970 (0.893 to 1.054)	0.471		NA	NA
**TB-BMD**						
MR Egger	9	1.010 (0.869 to 1.174)	0.901	4	1.012(0.957- 1.070)	0.714
Weighted median	9	0.998 (0.925 to 1.077)	0.962	4	0.994(0.962- 1.028)	0.739
MR PRESSO		NA	NA		NA	NA
**TB-BMD (age over 60)**						
MR Egger	9	0.929 (0.749 to 1.152)	0.523	4	1.036(0.941- 1.140)	0.546
Weighted median	9	0.919 (0.801 to 1.056)	0.235	4	0.983(0.928- 1.041)	0.557
MR PRESSO		NA	NA		NA	NA
**TB-BMD (age 45-60)**						
MR Egger	9	0.931 (0.777 to 1.114)	0.460	4	0.955(0.861- 1.059)	0.471

Weighted median	9	0.907 (0.784 to 1.048)	0.186	4	0.972(0.915- 1.032)	0.350
MR PRESSO		NA	NA		NA	NA
**TB-BMD (age 30-45)**						
MR Egger	9	1.310 (0.990 to 1.733)	0.100	4	1.035(0.852- 1.258)	0.761
Weighted median	9	1.214 (0.991 to 1.486)	0.061	4	1.028(0.943- 1.120)	0.534
MR PRESSO		NA	NA		NA	NA
**TB-BMD (age 15-30)**						
MR Egger	9	0.987 (0.682 to 1.430)	0.948	4	1.041(0.808- 1.340)	0.787
Weighted median	9	1.003 (0.742 to 1.355)	0.987	4	0.981(0.852- 1.129)	0.79
MR PRESSO		NA	NA		NA	NA
**TB-BMD (age 0-15)**						
MR Egger	9	1.042 (0.738 to 1.471)	0.823	4	1.030(0.907- 1.170)	0.695
Weighted median	9	0.977 (0.805 to 1.186)	0.812	4	1.016(0.941- 1.096)	0.693
MR PRESSO		NA	NA		NA	NA
**AF**						
MR Egger	8	0.992 (0.981 to 1.002)	0.177		NA	NA
Weighted median	8	0.998 (0.994 to 1.002)	0.400		NA	NA
MR PRESSO		NA	NA		NA	NA
**SF**						
MR Egger	3	1.005 (0.993 to 1.017)	0.573		NA	NA
Weighted median	3	0.999 (0.997 to 1.002)	0.692		NA	NA
MR PRESSO		NA	NA		NA	NA
**LF**						
MR Egger	7	1.003 (0.992 to 1.014)	0.645		NA	NA
Weighted median	7	1.000 (0.997 to 1.004)	0.864		NA	NA
MR PRESSO		NA	NA		NA	NA
**HF**						
MR Egger	9	0.999 (0.996 to 1.002)	0.449		NA	NA
Weighted median	9	0.999 (0.997 to 1.001)	0.330		NA	NA
MR PRESSO		NA	NA		NA	NA
**OPF**						

MR Egger	8	0.654 (0.164 to 2.611)	0.569	4	1.063(0.665- 1.698)	0.823
Weighted median	8	0.635 (0.271 to 1.490)	0.297	4	1.109(0.841- 1.463)	0.464
MR PRESSO		NA	NA		NA	NA
**OP**						
MR Egger	8	0.554 (0.320 to 0.958)	0.079	4	1.335(1.054- 1.690)	0.139
Weighted median	8	0.748 (0.462 to 1.212)	0.239	4	1.086(0.933- 1.264)	0.287
MR PRESSO		NA	NA		NA	NA

Results with *P* less than 0.05 highlighted in bold. “NA” indicates not applicable or no significant outliers. FA-BMD, forearm bone mineral density; LS-BMD, lumbar spine bone mineral density; FN-BMD, femur neck bone mineral density; eBMD, heel bone mineral density; TB-BMD, total body bone mineral density; AF, arm fractures; SF, spine fractures; LF, leg fractures; HF, heel fractures; OPF, osteoporosis fracture; OP, osteoporosis.

Supplementary Table 7 The MR-Egger intercept test results for circulating antioxidant metabolites.

Outcome	Exposure	MR Egger intercept	*P*
FA-BMD	α-tocopherol	0.035	0.184
LS-BMD	α-tocopherol	-0.004	0.803
FN-BMD	α-tocopherol	-0.009	0.473
eBMD	α-tocopherol	0.008	0.238
TB-BMD	α-tocopherol	-0.017	**0.049**
TB-BMD (age over 60)	α-tocopherol	-0.017	0.221
TB-BMD (age 45-60)	α-tocopherol	-0.013	0.363
TB-BMD (age 30-45)	α-tocopherol	-0.015	0.566
TB-BMD (age 15-30)	α-tocopherol	-0.006	0.847
TB-BMD (age 0-15)	α-tocopherol	-0.025	0.178
AF	α-tocopherol	0.000	0.373
LF	α-tocopherol	0.000	0.639
HF	α-tocopherol	0.000	0.534
OPF	α-tocopherol	0.062	0.402
OP	α-tocopherol	0.071	0.098
FA-BMD	Ascorbate	-0.004	0.769
LS-BMD	Ascorbate	-0.010	0.279
FN-BMD	Ascorbate	-0.009	0.238
eBMD	Ascorbate	0.005	0.250
TB-BMD	Ascorbate	-0.002	0.766
TB-BMD (age over 60)	Ascorbate	0.002	0.834
TB-BMD (age 45-60)	Ascorbate	-0.005	0.604
TB-BMD (age 30-45)	Ascorbate	-0.001	0.931
TB-BMD (age 15-30)	Ascorbate	-0.012	0.577
TB-BMD (age 0-15)	Ascorbate	-0.005	0.696
AF	Ascorbate	0.000	0.501
SF	Ascorbate	0.000	0.878
LF	Ascorbate	0.000	0.179
HF	Ascorbate	0.000	0.864
OPF	Ascorbate	-0.026	0.648
OP	Ascorbate	0.023	0.359
FA-BMD	γ-tocopherol	-0.004	0.757

LS-BMD	γ-tocopherol	-0.001	0.866
FN-BMD	γ-tocopherol	-0.001	0.925
eBMD	γ-tocopherol	0.004	0.125
TB-BMD	γ-tocopherol	-0.001	0.797
TB-BMD (age over 60)	γ-tocopherol	0.005	0.569
TB-BMD (age 45-60)	γ-tocopherol	-0.010	0.325
TB-BMD (age 30-45)	γ-tocopherol	0.005	0.681
TB-BMD (age 15-30)	γ-tocopherol	0.001	0.976
TB-BMD (age 0-15)	γ-tocopherol	-0.007	0.546
AF	γ-tocopherol	0.000	0.714
SF	γ-tocopherol	-0.001	0.270
LF	γ-tocopherol	0.000	0.058
HF	γ-tocopherol	0.000	0.659
OPF	γ-tocopherol	-0.088	0.080
OP	γ-tocopherol	0.007	0.767
FA-BMD	Retinol	0.015	0.312
LS-BMD	Retinol	-0.003	0.737
FN-BMD	Retinol	-0.001	0.902
eBMD	Retinol	-0.002	0.516
TB-BMD	Retinol	-0.008	0.169
TB-BMD (age over 60)	Retinol	-0.008	0.350
TB-BMD (age 45-60)	Retinol	0.001	0.938
TB-BMD (age 30-45)	Retinol	-0.010	0.499
TB-BMD (age 15-30)	Retinol	-0.009	0.672
TB-BMD (age 0-15)	Retinol	-0.015	0.232
AF	Retinol	0.000	0.839
SF	Retinol	0.000	0.861
LF	Retinol	0.000	0.798
HF	Retinol	0.000	0.111
OPF	Retinol	0.061	0.221
OP	Retinol	-0.016	0.564

Results with *P* less than 0.05 highlighted in bold. FA-BMD, forearm bone mineral density; LS-BMD, lumbar spine bone mineral density; FN-BMD, femur neck bone mineral density; eBMD, heel bone mineral density; TB-BMD, total body bone mineral density; AF, arm fractures; SF, spine fractures; LF, leg fractures; HF, heel fractures; OPF, osteoporosis fracture; OP, osteoporosis.

Supplementary Table 8 The complementary MR analyses results for circulating antioxidant metabolites.

MR methods	α-Tocopherol			γ-Tocopherol			Ascorbate			Retinol		
	No.SNPs	OR (95% CI)	*P*	No.SNPs	OR (95% CI)	*P*	No.SNPs	OR (95% CI)	*P*	No.SNPs	OR (95% CI)	*P*
**FA-BMD**												
MR Egger	9	0.368 (0.086 to 1.570)	0.219	13	1.188 (0.861 to 1.638)	0.317	10	1.216 (0.630 to 2.346)	0.576	21	0.986 (0.881 to 1.103)	0.806
Weighted median	9	1.076 (0.523 to 2.215)	0.842	13	1.187 (0.981 to 1.436)	0.078	10	1.034 (0.691 to 1.546)	0.872	21	1.053 (0.993 to 1.115)	0.083
MR PRESSO		NA	NA		NA	NA		NA	NA		NA	NA
**LS-BMD**												
MR Egger	8	1.490 (0.645 to 3.443)	0.386	13	1.053 (0.867 to 1.278)	0.612	9	1.279 (0.838 to 1.951)	0.291	18	1.010 (0.943 to 1.081)	0.781
Weighted median	8	1.202 (0.773 to 1.869)	0.413	13	1.044 (0.931 to 1.171)	0.465	9	1.031 (0.791 to 1.344)	0.822	18	0.997 (0.965 to 1.032)	0.883
MR PRESSO		NA	NA		NA	NA		NA	NA		NA	NA
**FN-BMD**												
MR Egger	8	1.419 (0.695 to 2.896)	0.373	13	1.052 (0.889 to 1.245)	0.567	9	1.192 (0.858 to 1.655)	0.330	18	1.004 (0.956 to 1.055)	0.873
Weighted median	8	1.014 (0.688 to 1.495)	0.943	13	1.023 (0.927 to 1.128)	0.657	9	0.992 (0.802 to 1.226)	0.940	18	1.001 (0.972 to 1.030)	0.959
MR PRESSO		NA	NA		NA	NA		NA	NA		NA	NA
**eBMD**												
MR Egger	9	0.851 (0.572 to 1.266)	0.452	13	0.945 (0.884 to 1.010)	0.123	11	0.970 (0.797 to 1.181)	0.769	20	1.003 (0.981 to 1.026)	0.795
Weighted median	9	1.010 (0.877 to 1.162)	0.895	13	0.987 (0.953 to 1.022)	0.465	11	1.036 (0.962 to 1.116)	0.347	20	1.000 (0.990 to 1.010)	0.968
MR PRESSO		1.007 (0.893 to 1.136)	0.910		1.019 (0.985 to 1.054)	0.283		1.038 (0.979 to 1.100)	0.213		0.998 (0.990 to 1.007)	0.684
**TB-BMD**												
MR Egger	9	1.861 (1.150 to 3.013)	0.039	13	1.012 (0.900 to 1.139)	0.841	11	0.904 (0.672 to 1.217)	0.524	23	1.022 (0.975 to 1.072)	0.376
Weighted median	9	1.148 (0.878 to 1.502)	0.313	13	1.019 (0.948 to 1.096)	0.610	11	0.891 (0.762 to 1.042)	0.148	23	0.993 (0.973 to 1.014)	0.529
MR PRESSO		NA	NA		NA	NA		NA	NA		NA	NA
**TB-BMD (age over 60)**												
MR Egger	9	1.684 (0.727 to 3.902)	0.263	13	0.934 (0.763 to 1.143)	0.520	11	0.831 (0.587 to 1.176)	0.323	23	1.030 (0.961 to 1.104)	0.412
Weighted median	9	0.977 (0.626 to 1.526)	0.919	13	0.986 (0.869 to 1.119)	0.831	11	0.869 (0.677 to 1.115)	0.269	23	0.992 (0.958 to 1.027)	0.643

MR PRESSO		NA	NA									
**TB-BMD (age 45-60)**												
MR Egger	9	2.327 (0.937 to 5.774)	0.111	13	1.125 (0.886 to 1.428)	0.355	11	1.058 (0.631 to 1.773)	0.836	23	0.990 (0.919 to 1.066)	0.789
Weighted median	9	1.598 (0.910 to 2.805)	0.103	13	1.038 (0.905 to 1.191)	0.594	11	0.824 (0.611 to 1.110)	0.203	23	1.003 (0.965 to 1.042)	0.887
MR PRESSO		NA	NA		NA	NA		NA	NA		NA	NA
**TB-BMD (age 30-45)**												
MR Egger	9	2.216 (0.432-11.362)	0.372	13	1.006 (0.736 to 1.373)	0.973	11	0.793 (0.446 to 1.413)	0.452	21	1.027 (0.933 to 1.131)	0.591
Weighted median	9	1.293 (0.604 to 2.766)	0.508	13	0.998 (0.829 to 1.201)	0.981	11	0.805 (0.565 to 1.149)	0.233	21	0.979 (0.933 to 1.026)	0.374
MR PRESSO		NA	NA		NA	NA		NA	NA		NA	NA
**TB-BMD (age 15-30)**												
MR Egger	9	1.960 (0.299-12.859)	0.506	13	0.973 (0.557 to 1.700)	0.926	11	0.967 (0.471 to 1.988)	0.930	22	1.039 (0.924 to 1.168)	0.529
Weighted median	9	1.332 (0.438 to 4.054)	0.613	13	1.104 (0.834 to 1.460)	0.490	11	0.954 (0.640 to 1.421)	0.816	22	1.019 (0.967 to 1.074)	0.480
MR PRESSO		NA	NA		NA	NA		NA	NA		NA	NA
**TB-BMD (age 0-15)**												
MR Egger	9	1.559 (0.520 to 4.680)	0.454	13	1.028 (0.794 to 1.331)	0.838	11	1.238 (0.437 to 3.507)	0.697	21	1.067 (0.901 to 1.264)	0.460
Weighted median	9	0.856 (0.452 to 1.621)	0.633	13	0.918 (0.778 to 1.083)	0.311	11	0.724 (0.378 to 1.386)	0.330	21	1.029 (0.943 to 1.123)	0.517
MR PRESSO		NA	NA		NA	NA		NA	NA		NA	NA
**AF**												
MR Egger	4	1.021 (0.991 to 1.052)	0.311	9	0.998 (0.993 to 1.003)	0.459	10	0.994 (0.975 to 1.013)	0.546	11	0.999 (0.996 to 1.003)	0.728
Weighted median	4	1.001 (0.985 to 1.018)	0.889	9	0.999 (0.996 to 1.002)	0.569	10	0.999 (0.992 to 1.006)	0.798	11	1.000 (0.999 to 1.001)	0.710
MR PRESSO		NA	NA		NA	NA		NA	NA		NA	NA
**SF**												
MR Egger		NA	NA	6	1.014 (0.993 to 1.036)	0.262	3	0.996 (0.977 to 1.014)	0.719	3	1.000 (0.995 to 1.005)	0.954
Weighted median		NA	NA	6	1.000 (0.998 to 1.002)	0.975	3	0.997 (0.992 to 1.002)	0.269	3	1.000 (0.999 to 1.000)	0.267
MR PRESSO		NA	NA		NA	NA		NA	NA		NA	NA
**LF**												

MR Egger	3	1.014 (0.988 to 1.040)	0.489	9	1.005 (1.000 to 1.009)	0.067	10	1.012 (1.000 to 1.024)	0.082	10	1.000 (0.997 to 1.003)	0.885
Weighted median	3	1.007 (0.994 to 1.019)	0.311	9	1.001 (0.999 to 1.004)	0.350	10	1.005 (0.999 to 1.010)	0.083	10	1.000 (0.999 to 1.001)	0.732
MR PRESSO		NA	NA		NA	NA		NA	NA		NA	NA
**HF**												
MR Egger	9	0.999 (0.984 to 1.014)	0.900	13	0.999 (0.996 to 1.003)	0.762	11	0.998 (0.991 to 1.005)	0.522	20	1.000 (1.000 to 1.001)	0.367
Weighted median	9	1.001 (0.993 to 1.009)	0.879	13	0.999 (0.997 to 1.001)	0.464	11	0.998 (0.994 to 1.002)	0.383	20	1.000 (0.999 to 1.000)	0.630
MR PRESSO		NA	NA		NA	NA		NA	NA		NA	NA
**OPF**												
MR Egger	9	0.262 (0.002-29.845)	0.597	13	2.029 (0.615 to 6.696)	0.270	11	4.217 (0.270-65.769)	0.331	20	0.799 (0.552 to 1.155)	0.248
Weighted median	9	1.195 (0.103-13.873)	0.887	13	0.876 (0.422 to 1.816)	0.721	11	2.126 (0.475 to 9.507)	0.324	20	0.958 (0.786 to 1.167)	0.667
MR PRESSO		NA	NA		NA	NA		NA	NA		NA	NA
**OP**												
MR Egger	9	0.205 (0.016 to 2.684)	0.266	13	0.913 (0.491 to 1.697)	0.779	11	0.792 (0.242 to 2.600)	0.710	20	1.100 (0.887 to 1.364)	0.395
Weighted median	9	1.192 (0.321 to 4.426)	0.793	13	0.984 (0.706 to 1.370)	0.923	11	0.869 (0.445 to 1.696)	0.681	20	1.051 (0.947 to 1.167)	0.349
MR PRESSO		NA	NA		NA	NA		NA	NA		NA	NA

Results with *P* less than 0.05 highlighted in bold. “NA” indicates not applicable or no significant outliers. FA-BMD, forearm bone mineral density; LS-BMD, lumbar spine bone mineral density; FN-BMD, femur neck bone mineral density; eBMD, heel bone mineral density; TB-BMD, total body bone mineral density; AF, arm fractures; SF, spine fractures; LF, leg fractures; HF, heel fractures; OPF, osteoporosis fracture; OP, osteoporosis.
